# Breaking It Down: Investigation of Binge Eating Components in Animal Models to Enhance Translation

**DOI:** 10.3389/fpsyt.2021.728535

**Published:** 2021-08-13

**Authors:** Britny A. Hildebrandt, Susanne E. Ahmari

**Affiliations:** ^1^Department of Psychiatry, University of Pittsburgh School of Medicine, Pittsburgh, PA, United States; ^2^Center for Neuroscience, University of Pittsburgh, Pittsburgh, PA, United States; ^3^Center for the Neural Basis of Cognition, University of Pittsburgh, Pittsburgh, PA, United States

**Keywords:** binge eating, eating disorders, animal models, feeding behavior, pre-clinical

## Abstract

Binge eating (BE) is a core eating disorder behavior that is present across nearly all eating disorder diagnoses (e. g., bulimia nervosa, binge eating disorder, anorexia nervosa binge/purge subtype), and is also widely present in the general population. Despite the prevalence of BE, limited treatment options exist and there are often high rates of relapse after treatment. There is evidence showing that genetic factors contribute to the heritability of BE and support for biological contributions to BE. However, more work is needed to fully understand neurobiological mechanisms underlying BE. One approach to target this problem is to separate BE into its distinct clinical components that can be more easily modeled using pre-clinical approaches. To date, a variety of animal models for BE have been used in pre-clinical studies; but there have been challenges translating this work to human BE. Here, we review these pre-clinical approaches by breaking them down into three clinically-significant component parts (1) consumption of a large amount of food; (2) food consumption within a short period of time; and (3) loss of control over eating. We propose that this rubric identifies the most frequently used and effective ways to model components of BE behavior using pre-clinical approaches with the strongest clinical relevance. Finally, we discuss how current pre-clinical models have been integrated with techniques using targeted neurobiological approaches and propose ways to improve translation of pre-clinical work to human investigations of BE that could enhance our understanding of BE behavior.

## Introduction

**Figure 1:**
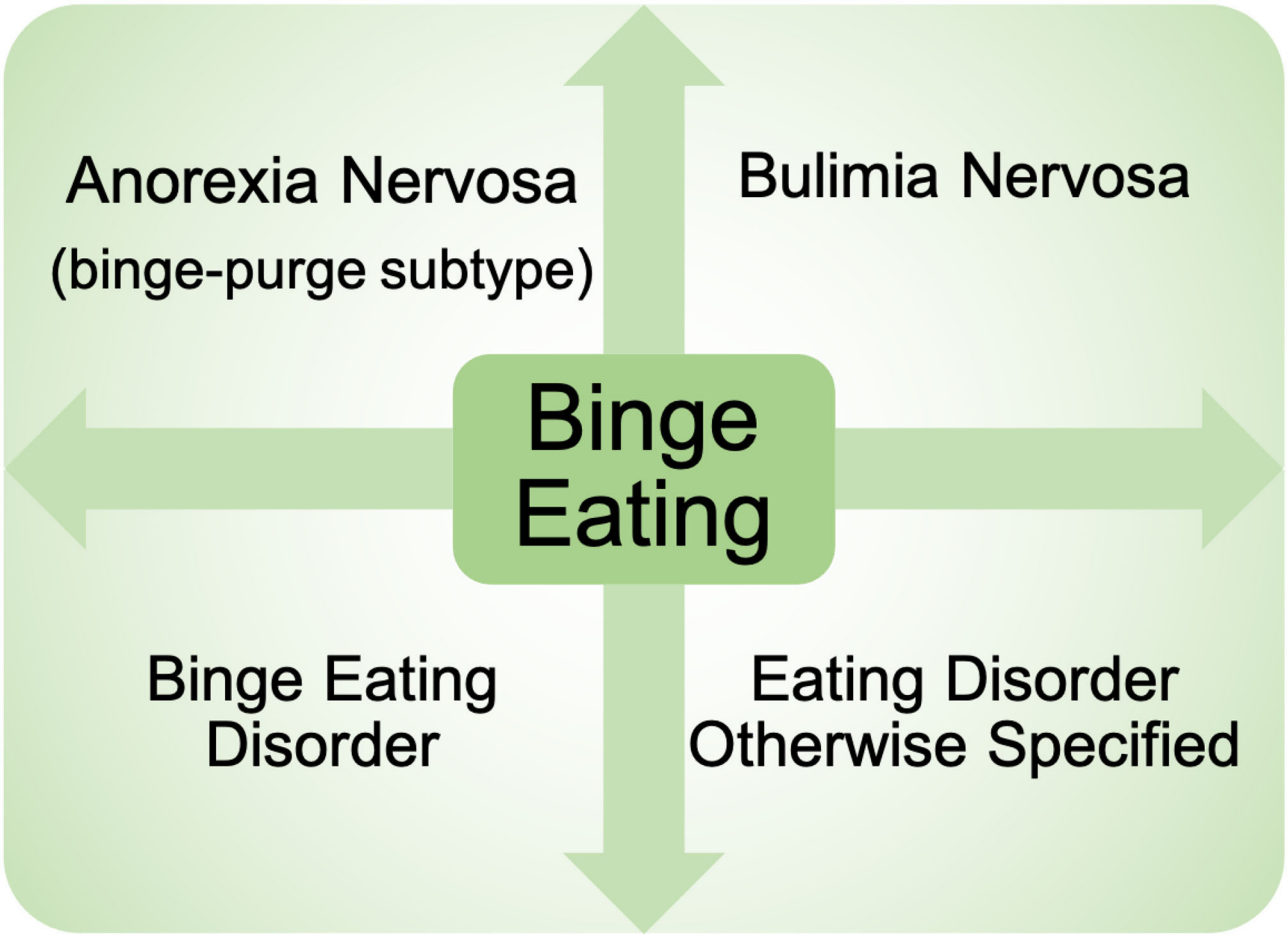
Presence of binge eating across eating disorder diagnoses. Binge eating is present across nearly all eating disorder diagnoses currently in the Diagnostic and Statistical Manual of Mental Health Disorders−5th Edition. Diagnoses include: binge eating disorder, bulimia nervosa, anorexia nervosa binge-purge subtype, as well as eating disorders otherwise specified.

Binge eating (BE) is a core eating disorder symptom that is present across nearly all eating disorder diagnoses (e.g., binge eating disorder, bulimia nervosa, anorexia nervosa—binge/purge subtype) (1) (Figure 1). The prevalence of BE disorder diagnoses continues to rise (2, 3), and rates of BE are highly prevalent in the general population (4, 5), highlighting the serious clinical impact of BE. BE is also associated with elevated rates of obesity (6–8), poor psychosocial outcomes (e.g., suicidal ideation) (9, 10), and significant medical consequences including type II diabetes and metabolic syndrome (11, 12). In addition, individuals with binge-related disorders often have high rates of relapse after treatment (13), leading to a significant impact on quality of life. Despite the substantial negative impact of BE, the etiology and mechanisms contributing to BE are still largely unknown.

Clinical studies of individuals with BE have provided evidence for neurobiological mechanisms underlying BE (14–16). For example, aberrant activity within circuitry associated with reward has been found in BE populations. Specifically, individuals with BE have a blunted response within the insular cortex during anticipation for a palatable food reward (sweet milkshake) (17) as well as the ventral striatum during monetary reward tasks (18, 19) compared to non-BE populations. Individuals with binge-related disorders also have increased activity within the medial orbitofrontal cortex, insular cortex, and anterior cingulate cortex in response to images of palatable food (20) compared to non-BE individuals. Additionally, individuals with BE have high levels of impulsivity (21, 22), and other studies have associated this with reduced activity in the prefrontal cortex during the Stroop task, a cognitive assessment of impulsivity which requires strong inhibitory control (23). The role of biological mechanisms underlying BE is further supported by studies in twins which have consistently shown that BE is a significantly heritable phenotype [~50% (24–26)], and studies which have found that diagnoses of BE disorder strongly aggregate within families (27). While this work has provided evidence for dysregulated neural activity and genetic mechanisms in BE, studies in humans are also complicated by a variety of factors such as comorbidity with other psychiatric conditions (9, 28) and psychosocial factors (29), which can make it challenging to fully disentangle patterns of neurobiological function that are specifically related to BE. However, pre-clinical approaches can isolate specific mechanisms underlying components of BE in the absence of these factors to provide additional insight into neural dynamics in BE.

**Figure 2:**
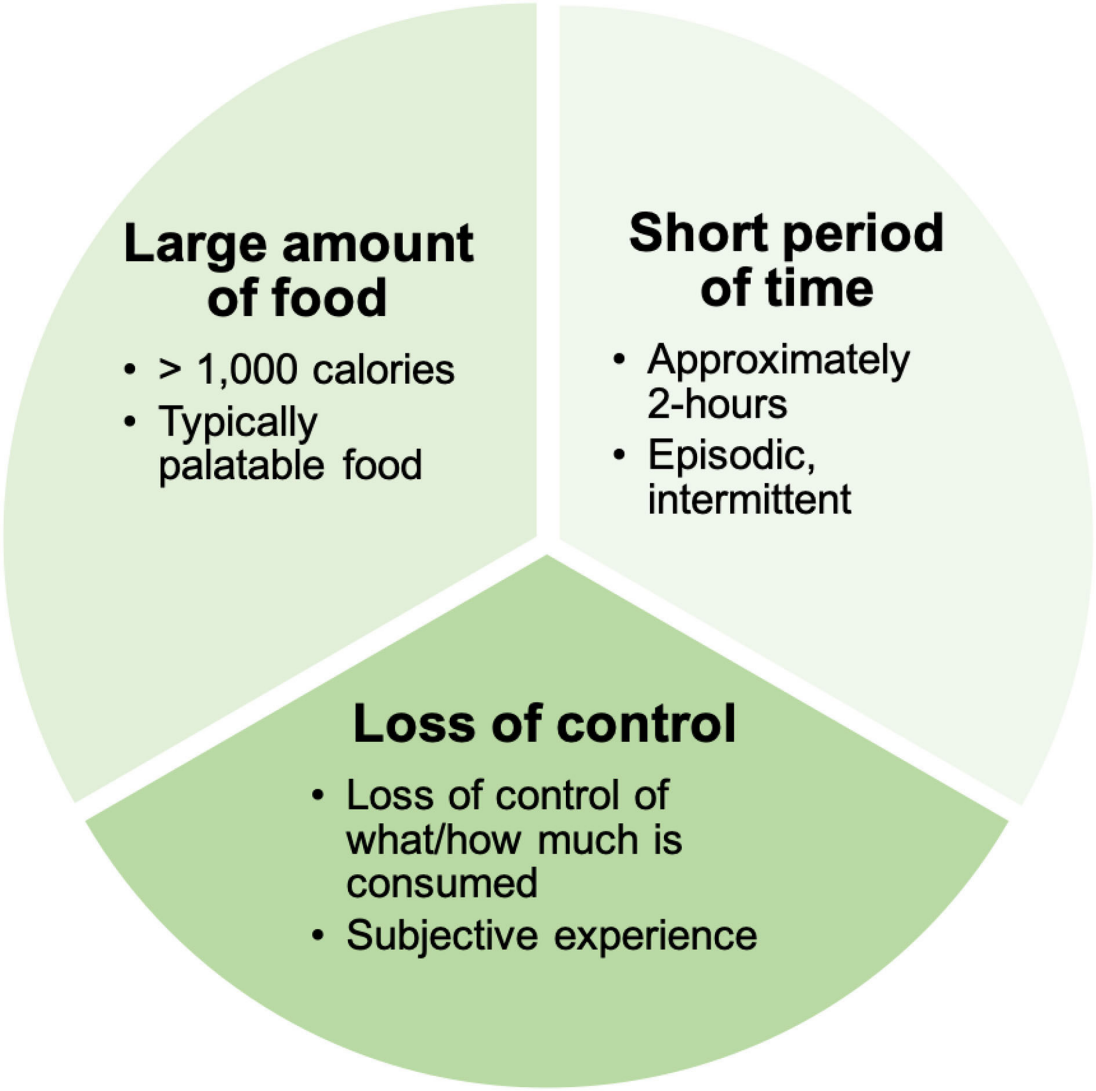
Clinical components of binge eating. Binge eating is comprised of three core clinical components: (1) The consumption of a large amount of food (that is typically palatable in nature). (2) The consumption of food is episodic and occurs within in a short period of time. (3) Individuals experience a loss of control over what and/or how much they are eating during binge eating episodes.

There has been a steady increase in the development and application of animal models to investigate mechanisms underlying BE (30–33), consistent with the increasing recognition of the impact of BE on quality of life. As with any animal model used to investigate psychiatric conditions, it is important to acknowledge that no pre-clinical approach can completely model complex human illnesses (34) like binge-related disorders. Nonetheless, pre-clinical approaches for BE can be used to isolate components of BE behavior that can be mechanistically dissected in animals. BE is comprised of three components according to criteria in the Diagnostic and Statistical Manual of Mental Disorders (1) (Figure 2). First, BE is associated with the consumption of objectively large amounts of food (1, 35). While not a diagnostic requirement, food consumed during BE is typically palatable food (PF), which is high in sweetness and fat, but low in nutritional value (36–39). Second, the large amounts of food are consumed during intermittent BE episodes that occur over a short amount of time–typically completed within 2 h according to diagnostic guidance (1), and consistent with observations in clinical populations (40). Third, individuals experience a loss of control over what and/or how much they are eating during these BE episodes (1). Loss of control is a subjective experience that is associated with significant levels of psychological distress (41), and is described by individuals that binge eat as the most salient or important feature of BE (42). By modeling these three core components of BE in animal models, we can dissect the circuits and specific cell populations underlying these BE constructs. This will provide novel information to improve our understanding of core BE components in humans and inform future clinical studies of BE.

**Figure 3:**
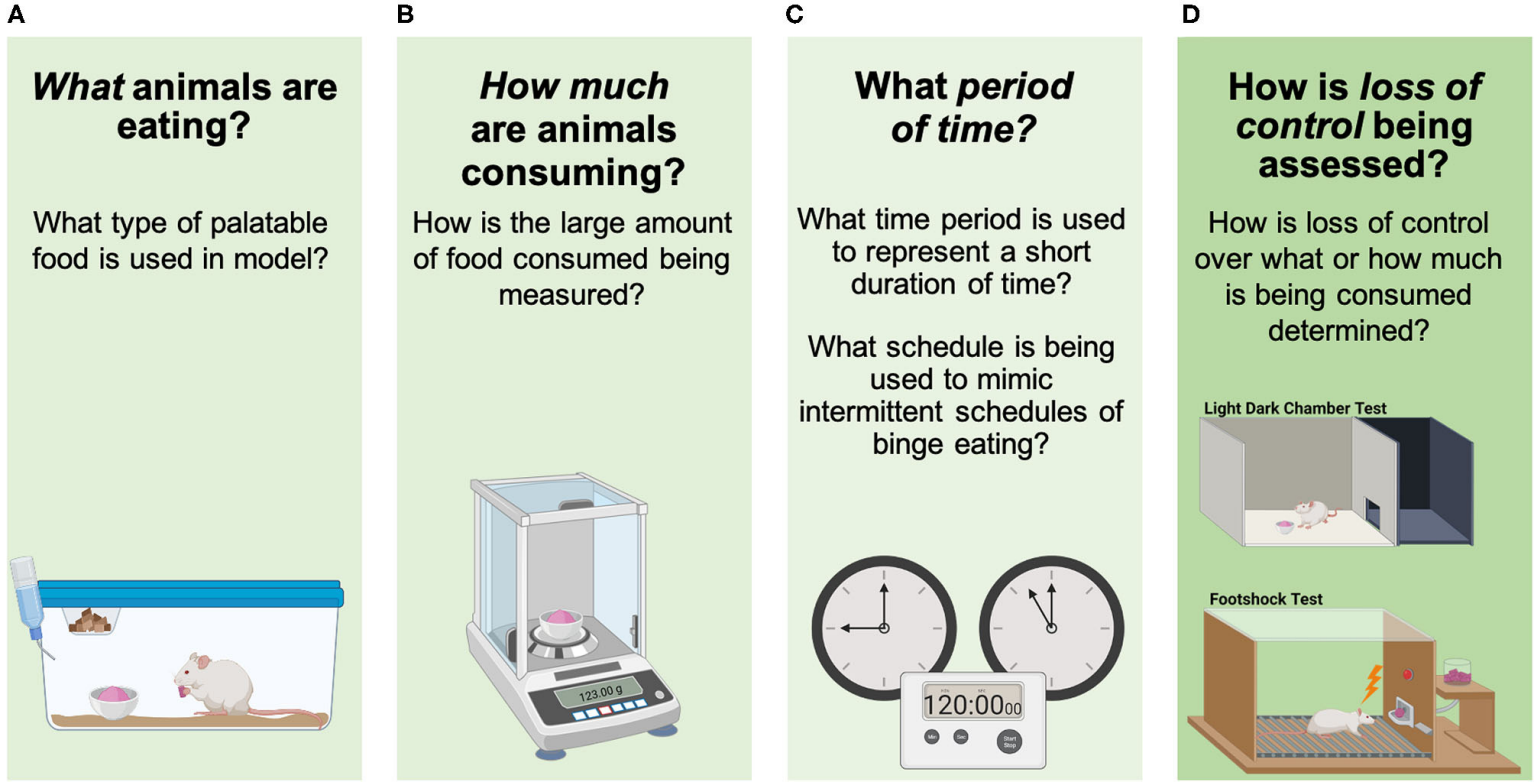
Assessing components of binge eating in animals. **(A)** What animals are eating in the model (i.e., what type of palatable substance is being used), **(B)** how much animals are consuming (i.e., how is an “objectively large amount of food” being defined), **(C)** what period of time is being used to capture a BE episode (i.e., intermittent/short period of time being used to examine BE), and **(D)** how loss of control over eating during BE is being determined (i.e., loss of control over what/how much is being consumed). Images were created with BioRender.com.

The overarching aim of this review is to evaluate how each of these three components of human BE behavior have been assessed using pre-clinical models (Figure 3). These include: *what* animals are eating in the model (i.e., what type of palatable substance is provided), *how much* animals are consuming (i.e., how is an “objectively large amount of food” being defined), what *period of time* is being used to capture a BE episode (i.e., what intermittent access schedule and short period of time is being used to examine BE), and how *loss of control* over eating during BE is being assessed (i.e., what approach is being used to assess loss of control over what/how much is being consumed). The goal of this review is not to identify one specific model that is best for examining circuit and cellular mechanisms underlying BE, but rather to break down previously used approaches into component parts that are translationally relevant for understanding clinical BE. Now, as new technologies become available that provide increased precision for monitoring and manipulating individual cell types and circuits, it is critical to evaluate which models for each BE component are best aligned with these methods to advance our understanding about how specific neurobiological factors contribute to BE.

## Overview of Approaches for Modeling Binge Eating in Animals

Over the last two decades, there has been an increase in the development of pre-clinical approaches to study BE (30, 33, 43, 44). These approaches have modeled many of the core clinical BE components listed above (Figure 2) and models have also been developed to examine other clinical characteristics related to BE such as the impact of caloric restriction and stress on BE. Notably, the following five models described are not intended to be a comprehensive review of all animal models for BE. These examples have been selected to highlight the progress in BE model development and provide a brief overview showing the diversity of approaches to modeling the core BE components in animals (Table 1). Subsequent sections of this review will further examine these models, along with other studies using animal models for BE, by breaking them down into the core components of BE (Figure 2) to directly examine how each component is modeled in animals (Figure 3).

**Table 1:** Component assessment of selected animal models for binge eating.

**Authors/Year**	***What*** **(palatable food used)**	***How much*** **(objectively large amount of food)**	**What *period of time* (access schedule)**	***Loss of control*** **(over what/how much)**
Boggiano et al. (45, 46), Klump et al. (47–50), Hildebrandt et al. (51, 52), LeMon et al. (53)	Sweet/Fat combination (Oreos, 4.8 kcal/g)	• Measured PF intake in grams • Classified animals as BE-prone/resistant based on average kcal intake	• 3–5 times per week • Measured intake at 1, 2, 4, and 24-h	Engagement in repeated foot shock to consume PF (54)
Cifani et al. (55), Di Bonaventura et al. (56, 57), Piccoli et al. (58), Romano et al. (59)	Sweet/Fat combination (Nutella, food pellets, water) (3.63 kcal/g)	• Measured PF intake in grams • Assessed intake in total kcal	• Measured intake after 2 and 24-h	Consumption of PF after exposure to a frustrative stress
Corwin and Wojnicki (60), Corwin et al. (61), Corwin (62), Wojnicki (63), Dimitriou et al. (64), Chawla et al. (65)	High fat (Crisco, 9.2 kcal/g)	• Measured total PF intake after exposure • Assessed intake in total kcal between groups	• Monday, Wednesday, Friday 2-h PF access • Daily 2-h PF access • Continuous chow only	–
Czyzyk et al. (66), Xu et al. (67), Cao et al. (68)	“High energy pellet” −40% fat, 16% sugar (4.3 kcal/g)	• Measured total PF intake at 2.5 and 24 hrs in grams • Assessed intake in total kcal between groups	• 1 24-hr exposure per week • *Ad libitum* chow and PF • *Ad libitum* chow	–
Halpern et al. (69), Doucette et al. (70), Sarica et al. (71)	High fat pellet (5.24 kcal/g)	• Assessed 1-h PF intake in kilocalories • BE criteria reached after consistent consumption of PF	• Daily 1-h PF access	–

*PF, palatable food; hr, hour; kcal, kilocalorie. Authors/Year column includes the surname of primary author from the original work that developed the model. Additional numerical citations indicate subsequent studies that have used or extended these models. Subsequent binge eating component columns include assessment of how each component was modeled in the original approach. The use of - in the loss of control column indicates that work using those models did not incorporate an assessment of loss of control in the model*.

### Corwin and Wojnicki—Intermittent Access to High Fat Palatable Food Elicits Binge-Like Eating

Some of the earliest pre-clinical investigations of BE highlighted the role of intermittent access to PF in the development of binge-like eating. Corwin and colleagues developed a model that provided direct support for the use of intermittent exposure to PF by examining the impact of different access schedules to a high fat PF (i.e., Crisco, “*what*,” Table 1) on binge-like eating (60, 61). Animals were divided into three groups: intermittent access (Monday, Wednesday, Friday, 2 h PF access), daily 2 h PF access, or a continuous chow only control (60, 61) (“*period of time*, ” Table 1). Animals with intermittent access (i.e., Monday, Wednesday, Friday for 2 h) had a higher total intake of PF than animals that received daily 2 h access (“*how much*,” Table 1), suggesting that more restricted or intermittent presentations of PF result in the most significant intake of PF reminiscent of binge-like eating (60–64). Further support for the role of intermittent PF access in the development of binge-like eating was found in another study that included an additional group of animals with continuous, non-intermittent access to PF. Here, intermittent access animals still consumed significantly more PF within the 2 h access period compared to continuous PF animals (64). Work using this model also confirmed that binge-like feeding was specific to PF and not standard chow by providing both PF and chow simultaneously. Animals on the intermittent and daily PF access schedules consumed more than 50% of their daily caloric intake on the high fat PF vs. chow (64, 65). Overall, work using this model provided foundational support for the importance of intermittent access to highly PF in eliciting binge-like eating in animals.

### Czyzyk et al.—Cyclic Access to Palatable Food Leads to the Development of Binge-Like Eating

The role of intermittent exposure to PF in the development of binge-like eating was extended to examine how weekly cycles of PF access impacted binge-like eating. Czyzyk et al. developed a model (66) that used a weekly, 7 day cycle comprised of 6 days of standard chow access followed by 1 day (24-h) of access to high fat, high sweetness PF (i.e., high energy pellet, “*what*,” Table 1). Intake was measured at two and a half hours and after 24-h on the PF access day (“*period of time*,” Table 1). Across study days, feeding in the weekly, cyclic PF access group was compared to animals with continuous access to PF and standard chow, and animals with continuous standard chow (66). After completion of at least 6–7 weeks of the cycle, only animals with intermittent, once weekly exposure to PF demonstrated a pattern of consistent binge-like eating (66, 67) as evidenced by the consumption of more than double the amount of kilocalories during the weekly 24-h PF exposure compared to animals in the continuous access groups (“*how much*,” Table 1) (66). Weekly, cyclic PF exposure animals displayed the majority of PF intake within the first two and a half hours of access, suggesting that these animals engaged in episodic consumption of PF, similar to human BE. This model has since been used in studies examining biological mechanisms underlying BE, in which ovariectomized female mice decreased binge-like eating after estrogen replacement (68), and that serotonin receptors are a potential treatment target to reduce binge-like eating (67). Together, findings demonstrate that this model of weekly, cyclic access to PF can be used to examine underlying biological mechanisms of binge-like eating.

### Halpern et al.—Consistency in PF Consumption to Identify a Chronic Binge Eating State

Diagnosis of many disorders characterized by BE (BE disorder, bulimia nervosa) requires that individuals engage in a consistent pattern of BE [i.e., at least one time per week for 3 months (1)]. Consistency in PF consumption has therefore also been used to characterize binge-like eating in a pre-clinical model for BE developed by Halpern and colleagues (69). In this model, all animals were equally exposed to high fat PF (i.e., high fat pellet, “*what*,” Table 1) daily for 1 h (“*period of time*,” Table 1). On this exposure schedule, animals consistently increased the amount of PF consumed across days (69). Binge-like eating was characterized as intake of PF of >25% of the caloric intake for the entire day during the 1 h exposure (69). Animals were considered to be in a chronic BE state once there was less than a 15% difference in PF intake between three consecutive PF exposures (“*period of time*,” Table 1), which occurred after seven exposures (69). Other work employing this model used a 2-h daily exposure to PF high in sugar and fat and a requirement of < 10% variability in PF intake across four consecutive feeding tests to establish chronic BE (70, 71). Animals successfully reached the chronic BE threshold after approximately eight exposures (70), similar to the original model (69). This approach to characterize chronic binge-like eating maximizes the sample size of BE animals in an experiment since all animals have the same PF exposure and threshold requirements to achieve BE. This model has been used for targeted neural manipulation to modify chronic BE. For example, Halpern and colleagues used this model to show that chronic BE animals receiving deep brain stimulation in the nucleus accumbens shell decreased binge-like eating compared to chronic BE animals receiving only sham surgery (69).

### Boggiano et al.—Binge Eating Resistant/Binge Eating Prone: Individual Differences in Rates of Binge Eating

The Binge Eating Resistant/Binge Eating Prone model, developed by Boggiano et al. (45), is an example of examining naturally occurring individual differences in propensity to engage in binge-like eating. To identify BE phenotypes, all rats in the paradigm were exposed to PF (Oreo cookies, “*what*,” Table 1) approximately three times per week, with at least 1 day in between exposures (45, 47, 48, 51, 52). PF was measured at multiple time points (1, 2, 4, and 24 h, “*period of time*,” Table 1). Animals were classified as BE-resistant or BE-prone using a tertile [or median split (45)] approach based on average PF intake across the paradigm. Animals falling consistently in the highest tertile of PF intake, and never in the bottom, are BE-prone (“*how much*,” Table 1). Conversely, animals consuming the lowest amounts of PF consistently across study days are labeled BE-resistant. Rates of BE proneness are ~20–30% of study populations (47, 72). Other work classifying animals as BE-prone and BE-resistant showed differences in feeding microstructure—i.e., BE-prone animals had significantly more licks compared to BE-resistant animals during a 1 h free access PF exposure (73). This may point to differences in motivation for PF such that BE-prone animals have higher motivation to consume PF compared to BE-resistant animals (73). During a 1 h PF access period after a stress-exposure, BE-prone animals had a shorter time to onset of the first lick, a higher rate of licking in the first minute of PF exposure, and shorter time between bouts compared to BE-resistant animals (73). These data may provide evidence for increased hedonic value of PF (73), specifically after stressful experience, in BE-prone animals.

The BE-Resistant/BE-Prone model has also been used to investigate other clinical outcomes and phenotypic characteristics of BE. Similar to human BE, there are strong sex differences in BE-prone phenotypes, with higher rates of BE-proneness among female rats vs. male rats in this model (47). Additionally, puberty is strongly linked to the emergence of BE in animals, similar to human BE in which puberty is a particularly risky phase of development for the onset of BE (48, 49, 74). There is also some evidence for compulsive behavior in BE-prone animals, such that they are more likely to engage in repeated and increasing foot shock to obtain PF compared to BE-resistant rats (“*loss of control*,” Table 1) (54). Together, these results support the BE-resistant/BE-prone model to examine individual differences in BE.

### Cifani et al.—Impact of Caloric Restriction and Stress on Binge Eating

Clinical studies have pointed to the combination of stress and caloric restriction as important risk factors for BE (75). An approach developed by Cifani et al. (55) targeted this clinical presentation of BE by combining a history of caloric restriction with stress to examine the impact on binge-like eating. Animals were food restricted to ~66% of their chow intake for 4 days (i.e., caloric restriction), then allowed to refeed on standard chow for 4 days (55). On the last day of the restriction/refeeding cycle, a mild, frustrative stress was administered, in which PF was placed outside of the feeding cage where it could be seen and smelled by animals, but not consumed for 15 min (55). This approach has been shown to increase corticosteroid levels in animals after 15 min, providing evidence for a stress response (55). The stressor was followed by 2 h (“*period of time*,” Table 1) of access to a high sweetness high fat PF (Nutella/food/water mixture, “*what*,” Table 1). A significant increase in PF intake during exposure (“*how much*,” Table 1) was observed in animals that experienced the combination of both a history of caloric restriction and stress compared to those with a history of only stress, only caloric restriction, or exposure to neither stress nor caloric restriction (55). A similar protocol using the forced swim test as the stressor prior to PF access also showed an increase in PF intake in animals with a history of caloric restriction compared to mice with caloric restriction and no stressor (76). Studies using this model have examined underlying biological mechanisms related to BE across estrus cycle phases, with binge-like eating occurring during the diestrus and proestrus phases and not the estrus phase (56). Another study showed that changes in cytokine levels within the hypothalamus during the estrous cycle were related to changes in binge-like eating (77). Additionally, previous work has used this model to identify potential candidates for pharmacological treatment of BE. Gavage administration of topiramate (55), systemic injections of corticotropin-releasing factor antagonists (57), gavage administration of an orexin receptor OX_1_R antagonist (58), and systemic oleoylethanolamide administration (59) all successfully reduced PF intake in animals with a history of caloric restriction and stress. Thus, this model for BE mimics a specific risk pathway to BE in which an increase in PF consumption is specific to animals with a history of caloric restriction and stress exposure, rather than dieting or stress alone.

## Breaking Down Models for be Into Component Parts

The five models discussed above demonstrate ways in which the core clinical components of BE (Figure 2) have been modeled in animals (Figure 3, Table 1). While each model has uniquely contributed to the establishment of pre-clinical approaches for studying BE, it is important to independently assess how each individual component of BE is represented within these models and in other pre-clinical studies of binge-like eating. This component assessment approach demonstrates the variety of modifications used in pre-clinical BE studies, including the variety of PF types used (“what” is being consumed during binge-like eating), different assessment approaches to determine how much PF is being consumed during binge-like eating (identification of “how much” by measuring amount of food consumed), and the range of PF exposure durations (over what short “period of time” is binge-like eating occurring). By considering the differences in how components are assessed in various animal models, we can identify which approaches most strongly map onto clinical BE in humans (Figure 2) to optimize future pre-clinical studies.

## Palatable Food Used (“What”)

BE episodes in humans involve the consumption of large amounts of PF (35). These foods tend to be high in sweetness and fat, but low in nutritional value (36–38). While this criterion is not a diagnostic requirement for BE (1), studies in humans have identified that BE episodes rarely contain non-PF (37, 38). Moreover, PF high in sweetness is shown to be preferred by BE populations and may predict BE frequency (78). While animal models for BE consistently use PF to elicit binge-like eating, the type of PF used is diverse, and different types of PF evoke different feeding responses from animals (33, 79).

### High Sweetness Palatable Food

High sweetness PF has been used across animal models for BE and is typically presented in the form of a liquid solution (e.g., sucrose, glucose) or high sugar pellet. Exposure to high sweetness PF can effectively evoke binge-like eating after exposures as short as 30 min and up to 24 h of access (80–87). However, there is evidence that the majority of high sweetness PF is consumed within the first hour of access, even if animals are provided longer (e.g., 12 h) access (88). There are individual differences in the consumption of high sweetness PF. The variability in PF intake allows for animals to be classified as BE-prone or BE-resistant based on amount of PF consumed (73, 89, 90) promoting the study of individual differences in BE phenotypes. Additionally, the caloric makeup of high sweetness PF does not impact binge-like eating, demonstrated by the fact that there were no differences in sucrose (caloric) or saccharin (non-caloric) consumption during a 4 h exposure (91). This finding suggests that palatability, rather than caloric value, is driving PF consumption.

### High Fat Palatable Food

Intermittent exposure to high fat PF also evokes binge-like eating in animals, with many similarities to models using high sweetness PF. High fat PF is typically presented in pellet or other solid (e.g., Crisco) forms. Binge-like eating on high fat PF is strongly related to intermittent access schedules, such that animals with intermittent exposure to high fat PF consume significantly more PF compared to animals with continuous access within the same timeframe (66, 67, 92). Animal models using high fat PF can successfully elicit binge-like eating over a short study duration (5–7 days) (68, 69) and caloric restriction is not needed to see binge-like eating (62). Animals exposed to high fat PF are able to consume a significant proportion of their total daily caloric intake during one to 2 h exposures (61, 63, 64, 93, 94). Additionally, intermittent binge-like eating on high fat PF has resulted in impairments in reversal learning (65) and changes to metabolic processes such as higher glucose and insulin levels (93) demonstrating the consequences of binge-like consumption. These findings reflect an aberrant eating pattern with profound effects similar to those seen in clinical populations, such that BE leads to impairments in cognitive tasks related to attentional bias (95) and an increased risk of developing metabolic syndrome (96).

### Comparison of High Sweetness vs. High Fat Palatable Food

Given similarities in the effects of exposure to high sweetness or high fat PF on binge-like eating, direct comparisons have been made to disentangle whether one PF has a stronger impact. Animals with access to either type of PF consumed a significant percentage of their daily caloric intake from PF [high sweetness (20–45%); high fat (55–86%)] (97, 98). While the greater percentage of daily consumption of high fat PF supports the notion that high fat may more strongly effect binge-like eating in animals (99), stronger effects of high sweetness PF have been found during continuous reinforcement operant training (FR1). Specifically, animals completed more lever presses for high sweetness PF compared to high fat PF (100). These discrepant findings may be related to the different paradigms used to assess binge-like behavior (i.e., free access schedule vs. operant training). Additionally, animals may be able to consume a larger amount of calories from high fat PF as this type of PF is higher in its caloric value per mass compared to high sweetness PF. Findings may also reflect differences in how the brain responds to specific types of PF. This idea is supported by a study indicating that systemic injections of baclofen, a GABA-B agonist, elicit different responses in PF consumption in rats depending on specific PF type, with reduced binge-like eating of high fat PF, and no effect on high sweetness BE (101). However, across studies there is evidence to suggest that both high sweetness PF and high fat PF can be effectively used to evoke BE-like feeding in animals.

### High Sweetness/High Fat Complex Palatable Food

More complex PF that is high in both sweetness and fat may provide more consistent findings and have stronger translational relevance to PF consumed during human BE episodes (36–38), as humans do not usually consume foods that are only high sugar or high fat. Indeed, complex PF developed for animals (e.g., pelleted formulas) has been used to successfully elicit binge-like eating after intermittent exposures ranging from 30 min to 2 h (70, 71, 102). Findings have been replicated using a variety of complex PFs including Nutella combined with food pellets and water (55–59, 77), Reeses Peanut Butter Drops and Nestle Chocolate Drops (103), Ensure drinks (104, 105), and sweetened condensed milk (106). Rates of BE-proneness are similar across studies using complex PF types high in both sweetness and fat such as Oreo cookies (45, 54) and vanilla frosting (47, 49, 51, 52). Therefore, while a range of PF has been used to successfully generate binge-like eating in animals, PF that is high in both sweetness and fat more strongly represents what is consumed during BE in humans and may best represent the core BE component of “what” type of food is being consumed.

## Measurement of Large Amount of Food (“How Much”)

BE episodes in humans are characterized by the consumption of an objectively large amount of food (1); however, the clinical classification is defined only by guidance suggesting “an amount of food that is definitely larger than what most people would eat in a similar period of time under similar circumstances” (1). Studies in humans have found that consumption >1,000 kcal is appropriate to characterize an objectively large BE episode, although the total amount consumed is often significantly higher (40, 107). However, assessing size of BE episodes in human populations is challenging due to inaccuracies in food recall and underreporting of total food intake (108). Pre-clinical approaches therefore have a distinct benefit over human studies of BE because intake of food consumed during binge-like eating in animals can be precisely measured by comparing total intake (in mass or milliliters) between PF access groups and controls (67, 88, 91). Additionally, examining the consistency of PF consumption or escalation of PF intake over time can highlight binge-like eating in animals (109), which is reflective of the consistency of BE required for diagnosis of binge-related disorders (i.e., one time per week for 3 months) (1).

### Total Caloric Amount Consumed (Kilocalories)

Pre-clinical approaches often measure PF consumed in grams and then convert this measurement into total kilocalories. The total caloric amount can be used to compare consumption differences across multiple food types (e.g., PF, chow, or both) (68, 98) and access schedules (e.g., intermittent, continuous) (64, 102, 104, 110). Inclusion of both total mass (in grams) and kilocalorie measurements within a study showed important differences in consumption, with mass highest for high sweetness PF, but larger total kilocalorie consumption of high fat PF (100). Total kilocalorie consumption has strong translational relevance given that kilocalorie assessments have been used in food recall assessments (111) and laboratory *ad libitum* test meal assessments (111) in human BE populations.

### Percent of Daily Calories

A key benefit of pre-clinical approaches for studying BE is the ability to accurately assess total food intake across not only the PF access period, but across an entire study day. This allows for important comparisons of intake between animals with intermittent binge-like eating and continuous access to PF. While research has shown that continuous PF access animals consume more total kilocalories within a day, animals with binge-like intermittent PF access consumed 44–85% of their total daily caloric intake within a 2 h access period (94, 97, 98, 102). These temporal differences in PF intake between continuous and intermittent access groups highlights important differences in how large amounts of PF are consumed. Since consumption of a large amount of food within a short period of time during intermittent access is similar to the episodic nature of human BE, it may trigger neural changes that are important to model in animals (Figure 2). There are also differences in percent of daily caloric intake consumed during different intermittent PF exposure schedules in a head-to-head study, such that animals with more restricted intermittent access (Monday/Wednesday/Friday, 2 h) consumed over 50% of their daily calories during exposure to PF compared to the 32% consumed by daily 2 h PF access (61). Further evidence for binge-like consumption during restricted intermittent access has also been observed after intermittent PF exposures as short as 10 min, resulting in consumption of nearly 43% of daily caloric intake (81). Together, these findings highlight that animals on intermittent access schedules engage in consumption of objectively large amounts of PF in a short duration of time, reflecting BE in humans.

### Consistent Consumption of Large Amounts of PF

Consistent engagement in BE is a requirement for diagnosis of most binge-related disorders (one time per week for 3 months) (1). Pre-clinical approaches for examining binge-like eating have targeted this clinical criterion by identifying animals that consume a large amount of food during PF access consistently across multiple exposures. Using a consistency of consumption threshold, animals that consumed high amounts of PF and consistently maintained intake of PF within 10–15% across consecutive days were identified as being in a chronic binge eating state (69–71). Groupings have also been made using statistical cutoffs (e.g., median split, tertiles) based on consistency of PF intake during a study period to identify extreme groups of BE-prone and BE-resistant animals (45, 48, 54, 65, 72, 73, 89, 90, 105). This allows for comparison between extreme BE and non-BE groups for analysis, which is translationally relevant to work comparing BE and non-BE human populations within a study (112, 113).

## Time Assessment (“Period of Time”)

BE is intermittent and episodic, with a beginning and end to each episode [“discrete” (1)]. In addition, diagnostic guidance suggests that BE episodes occur within a 2 h timeframe (1), and a 2 h duration has been reported in human BE populations as a frequent time in which a BE episode occurs (40). The intermittent PF access schedules used for eliciting binge-like eating in animals often model this 2-h duration, however, there has been success at triggering binge-like eating with other durations of intermittent PF exposure.

### Short-Duration Intermittent Access Schedules (Two Hours or Less)

A large number of pre-clinical studies of BE use intermittent access to PF for 2 h to elicit binge-like eating (55–62, 64, 70, 71, 86, 97, 103, 105, 106). Importantly, 2-h exposure to PF later in the dark phase, when animals are likely sated from standard chow consumption, still results in binge-like eating (93, 94, 98, 102), suggesting that animals will engage in binge-like eating that is beyond homeostatic needs. While 2 h access schedules align with diagnostic guidance for binge-related disorders (1, 40), shorter intermittent access periods of 1 h (33, 63, 65, 69, 73, 89, 90, 100), and as short as 10 min each day (81, 82), also result in significant consumption of PF in a short period of time.

### Long Intermittent Access Schedules (12–24 Hours)

The emergence of binge-like eating in animals has also been observed using intermittent schedules with longer PF exposures. Animals with intermittent 12 h access to PF dramatically increased their PF intake within the first hour of access (88). The binge-like eating evoked by this model was persistent after 2 weeks without PF access, shown by animals with a history of binge-like eating lever pressing for PF at a significantly higher rate than controls (114). Two days of PF access (i.e., 48 h) followed by 5 days of standard chow access also resulted in binge-like eating on PF exposure days (80, 83). Less frequent intermittent 24 h exposures occurring only 1 day per week also evoked binge-like eating such that animals consumed twice as many calories as continuous PF access animals during the exposure (66, 67), with the majority of the PF consumed within the first few hours (66). Similarly, evidence from other studies using longer access schedules shows that animals still consume the majority of PF during the first few hours of access (33, 66, 88). This highlights that while animals may have longer exposures to PF, consumption patterns are episodic and binge-like, occurring over a short duration of time.

### Comparison of Short vs. Long Access Schedules

Studies directly comparing short and long intermittent access schedules have found relatively consistent findings. Animals with 2, 4, or 8 h access to PF all increased PF intake across exposures (85), and assessments of PF intake at 1, 2, 3, and 24 h found that the majority of PF was consumed in the 4 h measurement (45, 54). Brief (30 min) vs. extended (24 h) intermittent PF exposures resulted in binge-like eating on both access schedules; however, the brief access animals consumed more PF within 30 min compared to the extended access group (84, 87). While both short and long access schedules may lead to binge-like eating, the majority of PF intake occurs within the initial hours of exposure. Therefore, PF exposure schedules of 2 h or less have strong evidence for binge-like eating in animals that also is highly reflective of BE episode duration in humans (40).

## Loss of Control

While most BE components can be modeled with relative ease, loss of control over eating provides a unique challenge for pre-clinical approaches. In humans, loss of control during BE is a subjective experience associated with psychological distress (41); and loss of control during BE has been identified as the most salient component to individuals engaging in BE, more so than the size or amount consumed during the episode (115, 116). The inherent subjectivity of this experience makes it much more difficult to conceptualize and assess in pre-clinical studies than more quantitative measures (33). Recent pre-clinical efforts have tried to address this challenge by probing loss of control using specific paradigms developed to assess compulsive behavior, which is linked to loss of control in BE (117). The compulsive-like behavior paradigms used in pre-clinical approaches determine if animals that engage in binge-like eating are more likely to endure punishment or negative outcomes (e.g., foot shock) in order to receive a palatable reward (117), indicating more compulsivity and/or increased motivation to consume PF which is reflective of loss of control relevant behavior. Specifically, animals with a history of intermittent access to PF demonstrate more willingness to experience foot shock (84, 86), endure repeated foot shocks increasing in intensity (54), and spend more time in the light side of a light-dark chamber where PF was presented compared to animals without a history of intermittent PF access (73, 89). BE-prone animals were also more likely to continue daily consumption of PF combined with lactose (which leads to physical discomfort) compared to BE-resistant animals that reduced PF + lactose intake over time (105). These findings suggest that animals with binge-like eating may have a higher likelihood of compulsive behavior, suggesting potential relevance to loss of control in BE.

Additionally, craving for PF may represent an additional loss of control relevant behavior. Previous work in humans has shown that higher levels of craving are associated with loss of control (118) and that craving mediates the relationship between addictive-like feeding behavior and BE (119). Food craving is also associated with loss of control eating in adolescents (120) and individuals with binge-related disorders experience higher levels of food craving compared to those that do not engage in BE (121). Therefore, assessments specifically related to craving may provide additional insight into loss of control relevant behavior using pre-clinical approaches. For example, animals with binge-like eating forced to abstain from PF intake are more likely to have increased rates of lever pressing for PF, which may suggest increased craving for PF after abstinence (122). Pavlovian conditioning paradigms aimed at associating environmental context cues with consumption of PF have shown increased consumption of PF, but not standard chow, suggesting a potential mechanism of cue-induced craving underlying increased PF intake (123). This may have relevance to how cues such as advertisements or social interactions could precipitate BE episodes in humans. Together, given the challenges of directly assessing loss of control in animals, it may be important to include multiple assessments of proxy behaviors (e.g., compulsivity, craving) to obtain a more comprehensive assessment of loss of control in BE when using pre-clinical models.

## Impact of Stress and Caloric Restriction

As reviewed above, pre-clinical approaches for examining BE have been successful at modeling the three core clinical components underlying BE: (1) consumption of a large amount of PF (2) in a short duration of time, and (3) loss of control over eating. Beyond these core components, other clinical characteristics of BE have been modeled in animals. While BE in response to stress is not listed among diagnostic criteria for binge-related disorders, there is evidence that stress and negative affect may increase food intake and BE in humans (124–128) and animals (73, 89, 90, 103). Additionally, caloric restriction has also been shown to increase the likelihood of BE in humans (129) and animals (130).

In animals, the combination of caloric restriction and stress elicits a strong BE response (131, 132). Animals with history of caloric restriction and stress (via foot shock) increased PF food intake compared to animals with only caloric restriction or only stress (132). Similar protocols found that other stressors, such as the forced swim test (76), a frustrative stress (55–59, 77), or the use of multiple stressors (e.g., wet bedding, novel noises, predator scent) (133) also led to binge-like eating in animals with a history of caloric restriction. It is important to caution that the increases in PF consumption in protocols involving caloric restriction may be driven by hunger (33), and overeating observed in human BE is not always driven by hunger (30). Therefore, while stress and caloric restriction are strongly linked to inducing binge-like eating in rodents, these models may be relevant only to a specific type of BE in humans.

## Controls and Comparison Groups For Pre-Clinical Studies of Binge Eating

Pre-clinical approaches for BE allow for careful selection of control and comparison groups. The ability to control food access on the levels of duration, amount, and type are not easily controlled in human studies, and highlight a unique strength of pre-clinical approaches. The most frequently used control for animals on a binge-like intermittent PF access schedule is animals with continuous access to standard chow diet and/or PF (60–64, 66–68, 80, 82, 84, 85, 87, 88, 92–94, 98, 102, 104). This design allows for comparisons aimed at understanding if any observed effects are due to the intermittent presentation of PF (binge-like access) or general PF consumption (non-intermittent PF access). Other approaches using individual differences to identify BE and control groups offer a more translatable approach to group selection. Here, animals are offered equal access to PF, similar to human exposure to PF. Groups are then identified based on the PF consumption during the paradigm (highest/consistent PF intake are BE, lowest/ consistent consumption of PF are non-BE/control group) (45, 48, 52, 72, 73, 89, 90, 105, 134), resulting in comparison of extreme groups of BE and non-BE animals.

Control groups for studies examining the impact of intervention or manipulation on BE use treatment or exposure specific controls (e.g., vehicle injection, sham surgery, stress/no stress), in which animals receive equal access to a specific PF, but the impact of intervention is determined by comparing binge-like eating outcomes between treatment groups (69, 70, 91) or after stress and/or caloric restriction (55–58, 76, 77, 103). Overall, while human studies are restricted in factors that can be controlled, pre-clinical approaches have the distinct benefit of limiting variability by isolating specific mechanisms of BE in control and comparison group selection.

## Discussion

BE is a core feature of eating disorders that is widely prevalent in the general population (135–137). However, there is still limited information regarding the neurobiological mechanisms that contribute to BE. Pre-clinical research has begun to fill this gap by using animal models for BE. There are unique benefits to using pre-clinical approaches to study BE; however, it is challenging to model all components of any psychiatric condition effectively in rodents (34). Here we have reviewed pre-clinical studies investigating BE and compared different approaches to modeling core components of BE (Figures 2, 3) including: what PF was used (“*what*”), how was a large amount of food representative of BE assessed (“*how much*”), how long did the access to PF last (“*period of time*”), and was there evidence of *loss of control* over eating during BE. Overall, there are important consistencies across studies, and these may highlight the most effective ways to model BE components to ensure translational relevance.

Identification of the most effective methods used to model BE components in animals will promote consistency across future studies, which will increase translational impact for clinical studies of BE. Broadly, PF has been used in all studies reviewed to model “*what”* type of food is being consumed during binge-like eating. However, complex PF that is high in sweetness and fat aligns with the types of PF consumed during human BE episodes (36–38). While work comparing high fat or complex PF to high sweetness PF showed binge-like eating across all PF types (97, 98, 110), complex PF evokes persistent binge-like eating with the strongest translational relevance to human BE, as humans do not typically consume foods that are exclusively high sugar or high fat.

Despite the challenges of assessing size of BE episodes and developing clear cutoffs for what is considered an objectively large amount of food in human BE (40, 107), “*how much*” food consumed during binge-like eating in animals is assessed using precise measurements of food intake. These measurements are taken to examine how much PF is being consumed, decide if it is an objectively large amount compared to control groups, and determine if PF intake comprises a significant proportion of total daily caloric consumption. The unique ability to make assessments of food intake at multiple time points highlights a relative strength for animal models for BE. Using these precise measurements, studies applying statistical cutoffs or thresholds related to consistent consumption of large amounts of PF to identify BE animals (45, 48, 54, 65, 69–73, 89, 90, 105) map strongly onto current diagnostic criteria requiring consistent BE (1) for diagnosis of binge-related disorders.

Similar to diagnostic guidance and human studies (1, 40), animal models for BE have most frequently used short intermittent PF access schedules to elicit binge eating (55–62, 64, 70, 71, 86, 93, 94, 97, 98, 102, 103, 105, 106) to capture a short “*period of time*.” Despite work using longer access durations, evidence shows that animals still consume the majority of PF during the first few hours of access (33, 66, 88), and that intermittent presentation of PF is key in modeling BE (43, 138). Therefore, animal models for BE can mimic diagnostic guidance in human BE by using 2-h intermittent PF exposure schedules to successfully evoke binge-like eating. Additionally, BE in humans occurs most frequently in the evening vs. morning (139). While animal models have successfully elicited binge-like eating at different points in the light cycle, studies presenting PF at later portions of the dark (active) phase [e.g., 4–8 h into the dark phase (98, 102, 110)] may provide additional translational relevance.

Finally, while “*loss of control*” is more challenging to assess in animal models for BE (33), studies have highlighted that animals with binge-like eating show more compulsive behavior for PF reward, suggesting that compulsivity paradigms are a useful proxy that is relevant to loss of control (54, 73, 84, 86, 89, 105, 117). However, much work remains to better conceptualize and assess translationally relevant loss of control in animal models for BE.

Animal models for BE that use components with strong translational relevance to human BE provide an excellent foundation for circuit level investigations, and research using targeted neurobiological approaches to study feeding behaviors is increasing (140, 141). Circuit manipulation techniques such as optogenetics (84, 141) and chemogenetics (92) are excellent tools to identify the role of specific neuronal projections or cell populations during BE behaviors. Given the potential role of reward related circuitry in BE (15), work using these techniques has begun to target specific neuronal projections within these circuits to modify binge-like eating behavior. Optogenetic inhibition of projections from the insula to the nucleus accumbens, brain regions strongly associated with taste and reward, respectively, reduced compulsive responding for palatable rewards during an operant task in animals with binge-like eating (84). Similarly, chemogenetic inhibition of specific prefrontal cortical projections to the nucleus accumbens decreased impulsive behavior measured by a serial reaction task in animals with binge-like eating (92). Application of deep brain stimulation to the nucleus accumbens or prefrontal cortex also successfully reduced binge-like eating behavior in animals (69–71, 142, 143). This work provides important information implicating activity in these regions—particularly the nucleus accumbens—as drivers of aberrant eating behavior, highlighting potential targets for clinical intervention.

Pre-clinical approaches combined with targeted circuit and cellular level techniques have the potential to deeply impact our understanding of the neurobiological mechanisms underlying of BE. Therefore, as more techniques become available for studying circuit dynamics in animals, we suggest using a component assessment approach (see Table 1 as an example) during the first steps of study design. With this strategy, researchers can first clearly identify the specific ways in which their work will model core components of BE (Figures 1, 2). The Research Domain Criteria (RDoC) approach (144) provides a precedent for using such a component assessment approach to break down complex psychiatric illness into manageable pieces for investigation. The RDoC framework is an excellent approach for understanding eating disorders (145), particularly BE behavior, as it cuts across multiple dimensions of eating disorder diagnoses (Figure 1). After assessing how BE components will be modeled, circuit and cellular level techniques, currently unavailable in human research, can then be used to investigate the selected components in BE and non-BE animals. After experiment completion, explicit discussion of the component assessment approach should be included in publication and dissemination of pre-clinical findings. This will promote clarity of approach for potential replication studies, while also providing important information for clinical investigators to translate circuit level findings in animals to study development in clinical BE samples.

In sum, animal models for BE are an essential tool for understanding neurobiological mechanisms underlying BE and binge-related eating disorders. A component assessment approach based on the work reviewed here will increase the utility of translation between pre-clinical and clinical studies, advancing our understanding of BE and allowing us to work toward development of targeted and effective interventions.

## Author Contributions

BH conceptualized and drafted the work. SA reviewed the work. All authors contributed to the article and approved the submitted version.

## Conflict of Interest

The authors declare that the research was conducted in the absence of any commercial or financial relationships that could be construed as a potential conflict of interest.

## Publisher's Note

All claims expressed in this article are solely those of the authors and do not necessarily represent those of their affiliated organizations, or those of the publisher, the editors and the reviewers. Any product that may be evaluated in this article, or claim that may be made by its manufacturer, is not guaranteed or endorsed by the publisher.
